# The area ratio of Modic changes has predictive value for postoperative surgical site infection in lumbar spine surgery: a retrospective study

**DOI:** 10.1186/s12891-024-07257-9

**Published:** 2024-02-13

**Authors:** Yanhang Liu, Qian Chen, Yueran Wang, Jiangtao He

**Affiliations:** Department of Orthopedics, Affiliated Hospital of North Sichuan Medical College, Nanchong, 637500 Sichuan China

**Keywords:** Modic changes, Lumbar spine surgery, Surgical site infection, Predictive diagnosis, Total endplate score

## Abstract

**Background:**

Increasing evidence suggests an association between Modic changes (MC) and subclinical infection and inflammatory reactions. However, the relationship between preoperative MC and surgical site infection (SSI) has not been fully explored. This study aims to investigate the correlation between MC and SSI.

**Methods:**

A retrospective analysis was conducted on patients (*n* = 646) who underwent single-level lumbar spine surgery for lower back pain in our hospital between 2018 and 2023. According to the Centers for Disease Control and Prevention (CDC) criteria, the patients were divided into an SSI group (*n* = 40) and a Non-SSI group (*n* = 606). Univariate analysis was performed to determine the statistical differences in variables between the two groups, and the variables with significant differences were included in a multivariable logistic regression analysis to identify independent risk factors for SSI. Receiver operating characteristic (ROC) curve analysis was performed on the independent risk factors.

**Results:**

The SSI group and the Non-SSI group exhibited significant differences in diabetes prevalence, MC prevalence, Total endplate score (TEPS) and area ratio of MC (*P* < 0.05). Age, gender, American Society of Anesthesiologists(ASA)score, hypertension, coronary heart disease (CHD), chronic obstructive pulmonary disease (COPD), MC classification, and the location of MC in the endplate showed no significant differences (*P* > 0.05). Multivariate binary logistic regression analysis was performed on the variables with significant differences, and the results indicated a significant correlation between TEPS (*P* = 0.009) and the area ratio of MC changes (*P* = 0.001) with SSI. ROC curve analysis was performed on the TEPS and area ratio of MC changes, and the results showed that the diagnostic value of TEPS (AUC: 0.641; CI: 0.522–0.759) is lower than the area ratio of MC (AUC: 0.722; CI: 0.621–0.824), and the combined diagnosis did not significantly improve the diagnostic value (AUC: 0.747; CI: 0.653–0.842). The area ratio of MC had moderate diagnostic value for SSI (AUC: 0.722; CI: 0.621–0.824), with a cut-off value of 24.62% determined by the Youden index (sensitivity: 69.2%; specificity: 73.1%), and for every 1% increase in the area ratio of MC changes, the risk of SSI in MC patients increased by 10.3% (OR = 1.103; CI: 1.044–1.167).

**Conclusion:**

The area ratio MC and the TEPS are independent risk factors for SSI after lumbar spine surgery. The predictive value of the area ratio of MC is greater than TEPS, and when the two are combined, the predictive value is not significantly improved. When the rate of MC exceeds 24.62%, caution should be exercised regarding the occurrence of SSI.

## Background

Modic changes (MC) refer to visible signal alterations in the vertebral endplates and bone marrow observed on magnetic resonance imaging (MRI). These changes were initially reported by De Roos et al., who described signal variations near the vertebral endplates on MRI scans [1]. Radiologist Michael Modic later provided a description and classification of MC. MC is categorized into three types: Type 1 appears as a low signal area on T1-weighted images (T1WI) and a high signal area on T2-weighted images (T2WI). Pathologically, it is characterized by inflammatory changes, with fibrous tissue replacing the normal bone trabeculae, along with endplate fissures, destruction, and proliferation of vascularized granulation tissue beneath the cartilage. Type 2 appears as high signal areas on both T1WI and T2WI and represent fatty replacement of normal bone marrow. Type 3 appears as low signal areas on both T1WI and T2WI, it is characterized by replacement of bone marrow fat with sclerotic bone [2, 3].

MC is considered an independent prognostic factor for the outcome of lumbar spine surgery, indicating poor clinical results. It has been reported that the prevalence of MC in patients with lower back pain is approximately 40–50%, compared to 6% in the general population [4–6]. The underlying mechanism is still controversial, with previous research suggesting that biomechanical causes are an important etiology [7, 8]. However, an increasing number of studies have found associations between MC and inflammatory reactions and subclinical infections. Researchers have cultured Propionibacterium acnes (P. acnes), a bacterium associated with acne and dental abscesses, from intervertebral discs of MC patients or animal models [9, 10]. Injection of P. acnes directly into healthy rabbit intervertebral discs has also been shown to induce MC [11]. Dudli et al. found that metabolites and byproducts released by P. acnes in intervertebral discs of MC patients elevate levels of pro-inflammatory cytokines (including interleukin-6, IL-1β, TNF-α, prostaglandins, etc.) in the body [12–14], and inflammatory markers (including neutrophils, CRP, ESR, etc.) are significantly elevated in MC patients [15, 16]. These studies suggest the presence of subclinical infection and a systemic inflammatory response in individuals with Modic changes. This further raises doubts about whether the presence of MC increases the risk of postoperative surgical site infection (SSI). Recently, Rajasekaran et al. found that Presence of Modic type 1 change increases risk of postoperative pyogenic discitis following decompression surgery for lumbar canal stenosis [17]; Pradip et al. found that presence of preoperative MC and total endplate score (TEPS) were independent risk factors for surgical site infection [18]. These studies further confirm the previous hypothesis.

The cartilage endplate, supported by a bony endplate, is responsible for anatomical integrity and controls diffusion, which is the only source of nutrients for the intervertebral disc [19, 20]. Rupture of the endplate establishes disc-to-bone marrow contact and may lead to severe autoimmune inflammation and may also lead to disc neovascularization and destruction [21]. We hypothesize that MC may cause disturbances in the structure and function of the cartilage endplate, thereby increasing the risk of SSI. However, the correlation between MC and SSI is currently limited and controversial. Therefore, this study explores in detail the correlation between MC (including presence, type, location, area, TEPS) and SSI, and quantified the severity of MC with MRI, defined as the area ratio of MC. The area ratio of MC and TEPS were used to predict SSI, which provided a reference for orthopedic surgeons to assess the risk of SSI in MC patients.

## Methods

### Participants

**Fig. 1:**
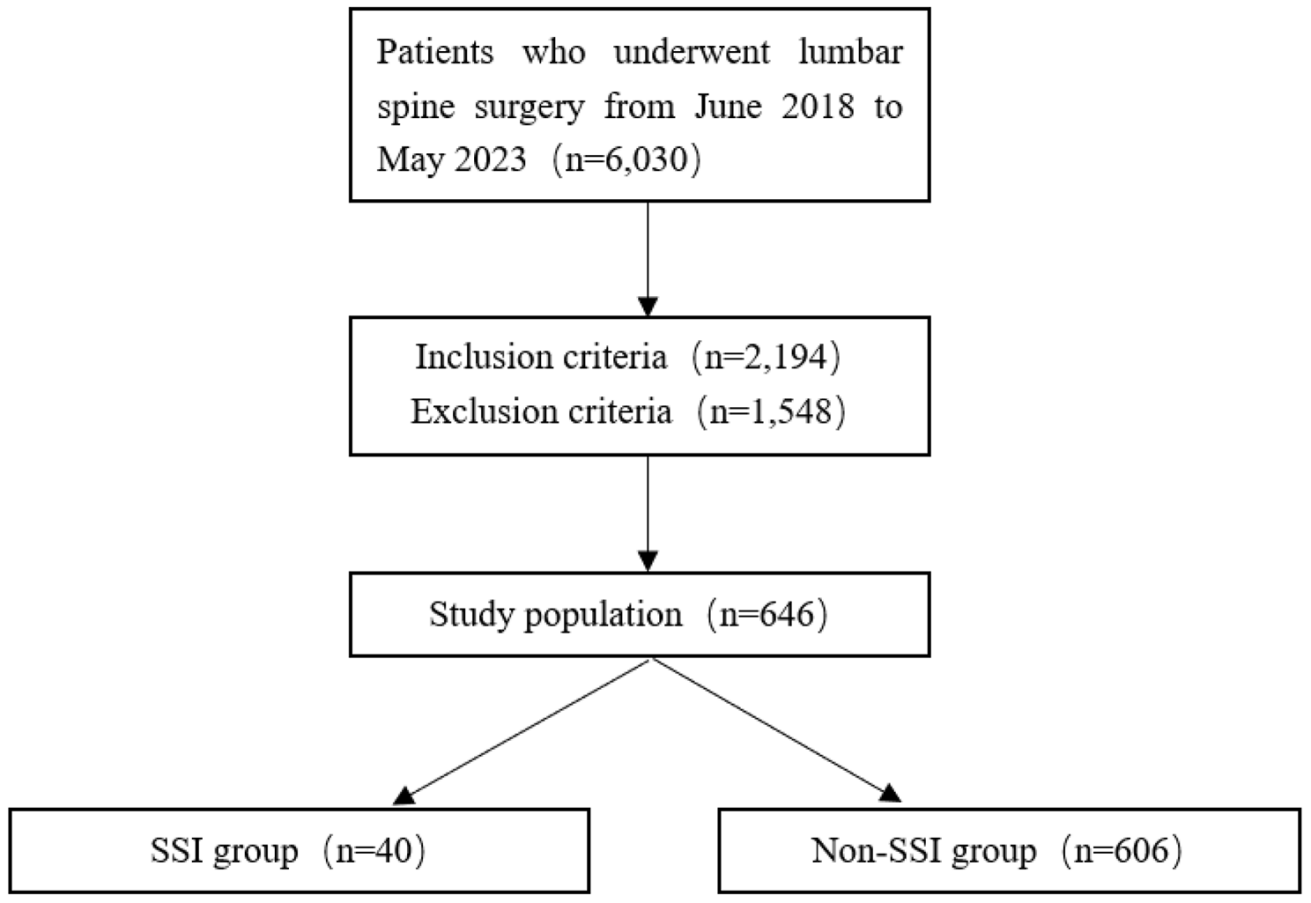
The sample allocation details

After obtaining approval from the Institutional Review Board (Protocol number: 2023ER89-1), we conducted a retrospective case-control study on 6,030 patients who underwent lumbar spine surgery at our hospital from June 2018 to May 2023. After the screening process, a total of 646 patients were included (Fig. 1). According to the Centers for Disease Control and Prevention (CDC) definition of SSI [22], the patients who were followed up for 90 days after surgery were divided into an SSI group (*n* = 40) and Non-SSI group (*n* = 606). Inclusion criteria were as follows: (1) patients with preoperative lower back pain who underwent lumbar spine surgery; (2) all lumbar spine surgeries were single-level procedures; (3) BMI between 18.5 and 24. Exclusion criteria were as follows: (1) patients with chronic inflammatory diseases or autoimmune deficiency diseases; (2) long-term use of immunosuppressive agents, steroids, or antibiotics; (3) long-term smoking or alcohol consumption; (4) concomitant diseases such as lumbar vertebral fracture, infection, or tumor; (5) revision surgery; (6) incomplete clinical data.

### Data collection

**Fig. 2:**
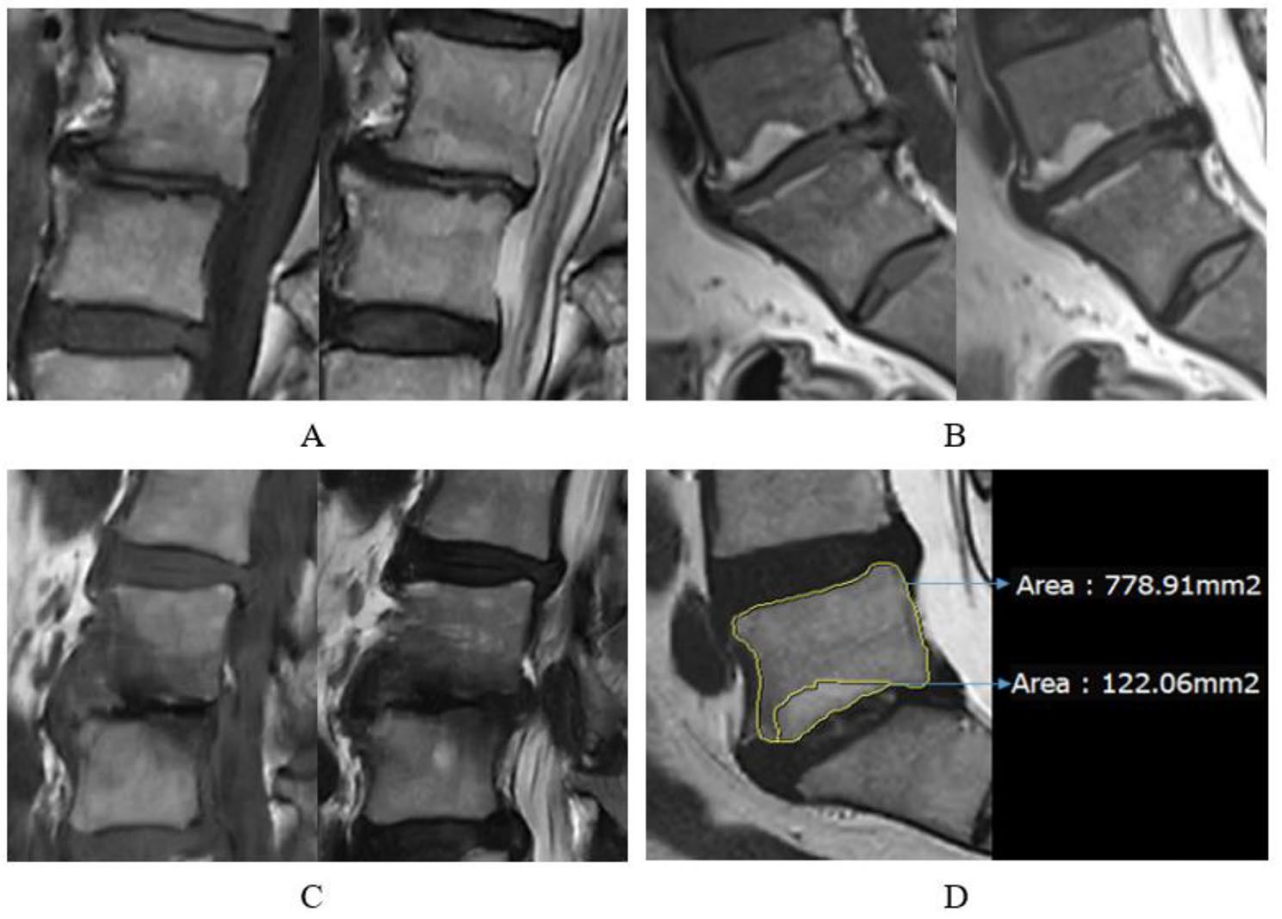
**A**: Type 1 MC, **B**: Type 2 MC, **C**: Type 3 MC, **D**: The area ratio of MC (%) is defined as the area of MC in the sagittal plane (mm²) divided by the area of the vertebral body (mm²), and the result is rounded to two decimal places

We collected basic patient information, including gender, age, American Society of Anesthesiologists(ASA)score, and comorbidities (hypertension, diabetes, chronic obstructive pulmonary disease, and coronary heart disease) from the electronic medical records of our hospital. We analyzed MRI of the upper and lower endplates of discs at surgical level in 646 patients, and based on the signal characteristics on T1-weighted imaging (T1WI) and T2-weighted imaging (T2WI), we diagnosed them with Type 1 MC (Fig. 2A) when T1WI showed low signal and T2WI showed high signal, Type 2 MC (Fig. 2B) when T1WI showed high signal and T2WI showed equal or slightly higher signal, and Type 3 MC (Fig. 2C) when both T1WI and T2WI showed low signal. The measurement of the area ratio of MC was performed by calculating the ratio of the maximum area of Modic changes to the vertebral body area on the sagittal plane of the MRI images (Fig. 2D). If there is MC at both the upper and lower endplates of discs, take the sum of the area ratio. All area measurements were conducted using the software provided by our hospital’s PACS system. TEPS was rated according to the criteria proposed by Rajasekaran et al. (Table 1).

**Table 1:** The total endplate score (TEPS) is calculated as a sum of the individual endplate scores adjacent to a disc with a score ranging from 2 to 12 for each disc level

MRI characteristics	score
No EP break or defects; Uniform hypointense band; Symmetrically concave; No MC	1
Focal thinning of the EP; No EP breaks; No MC	2
Focal disc marrow contacts; Normal contour of EP maintained; No MC	3
Defect up to 25% of width of EP; Typical depression present; MC usually present	4
Defect up to 50% of width of EP; Typical depression present; MC usually present	5
Complete EP damage; Irregularity and sclerosis of EP; MC usually present	6

All MRI scans were independently evaluated by two radiologists with over 10 years of experience who were unaware of patient information. The evaluation of both includes the use of MRI to assess the presence, types, locations, area ratio of MC and TEPS (kappa > 0.8). The area ratio is taken as the average of the two. For cases with obscure signal intensity boundaries, two radiologists repeat the measurement three times and take the average. Then, the average of the two is further taken as the final MC area. For special positional distributions, this study assumes that the anterior 2/3 MC is anterior and the posterior 2/3 MC is posterior. There is no MC at the edges of the anterior and posterior regions as the central type, and there is a distribution of MC in the anterior, central, and posterior regions as the entire type. After detailed discussions, the two observers reached a consensus on differences in the presence, types, locations of MC and differences in TEPS.

### Data analysis

Use Power Analysis to determine the minimum sample size for this experimental design. Compare the mean and standard deviation between the SSI and Non-SSI groups based on TEPS values. The mean of the SSI group was 6.725, with a standard deviation of 2.364, and the mean of the Non-SSI group was 5.112, with a standard deviation of 2.235. The Cohen’s d effect size was calculated to be 0.719. The efficacy analysis showed that in order to achieve a Cohen’s d effect size of 0.719 (which is a medium to large effect size), at a significance level of 0.05 and an 80% efficacy, each group needs at least 31 samples.

Quantitative variables following a normal distribution were expressed as mean ± standard deviation and assessed using independent sample t-tests. Qualitative variables were evaluated using chi-square tests or Fisher’s exact tests. Binary logistic regression analysis was conducted for variables showing significant differences. Quantitative variables with independent risk factors were analyzed using ROC curves, and the optimal cutoff value was determined using the Youden index. A *p*-value less than 0.05 was considered statistically significant.

## Results

### Main characteristics of patients

The SSI group and the Non-SSI group exhibited significant differences (*P* < 0.05) in diabetes prevalence, MC prevalence, TEPS and area ratio of MC. However, there were no significant differences in age, gender, ASA score, comorbidities, MC classification, and the location of MC in the endplates between the two groups (Table 2).

**Table 2:** Difference analysis of general data

Variables	SSI(*n* = 40)	Non-SSI(*n* = 606)	*P*
Age (years)	60.7 ± 10.01	58.7 ± 11.08	0.266
Gender(male/female)	22/18	311/295	0.652
ASA score(I-II/III-V)	31/9	476/130	0.876
Hypertension(yes/no)	10/30	124/482	0.493
Diabetes (yes/no)	11/29	91/515	**0.036**
COPD (yes/no)	4/36	76/530	0.822
CHD (yes/no)	2/38	47/559	0.742
Rate of MC (%)	27.38 ± 7.06	21.95 ± 6.90	**0.000**
MC (yes/no)	26/14	279/327	**0.022**
TEPS	6.73 ± 2.36	5.11 ± 2.24	**0.000**
Types of MC			0.537
MC1	11	110	
MC2	14	139	
MC3	1	30	
Location of MC			0.667
Anterior	9	105	
Central	0	8	
Posterior	1	20	
Entire	16	146	

Bold text denotes statistical significance

### Univariable and multivariate logistic regression analysis of risk factors for SSI

Univariate binary logistic regression analysis was conducted on variables with significant differences, and it was found that diabetes prevalence, MC prevalence, TEPS and area ratio of MC were significantly correlated with SSI(*P*<0.05). The variables with significant differences were subjected to multifactorial binary logistic regression to eliminate mutual interference among the factors. The results indicated a significant correlation between TEPS (*P* = 0.009) and area ratio of MC (*P* = 0.001) with SSI. For every 1 score increase in TEPS, the risk of SSI increases by 38.2% (OR = 1.382; CI: 1.085–1.759); For every 1% increase in area ratio of MC, the risk of SSI in MC patients increased by 10.3% (OR = 1.103; CI: 1.044–1.167) (Table 3).

**Table 3:** Univariable and multivariate logistic regression analysis of risk factors for SSI

Variable	Univariate logistic analysis		Multivariate logistic analysis	
	Odds ratio (95%CI)	*P* value	Odds ratio (95%CI)	*P* value
Area ratio of MC	1.106 (1.047–1.168)	**0.000**	1.103 (1.044–1.167)	**0.001**
MC	2.177 (1.115–4.250)	**0.023**	1.968 (0.992–3.906)	0.053
TEPS	1.350 (1.173–1.553)	**0.000**	1.382 (1.085–1.759)	**0.009**
Diabetes	2.147 (1.036–4.450)	**0.040**	0.376 (0.107–1.322)	0.127

Bold text denotes statistical significance

### ROC curve analysis TEPS and area ratio of MC

**Fig. 3:**
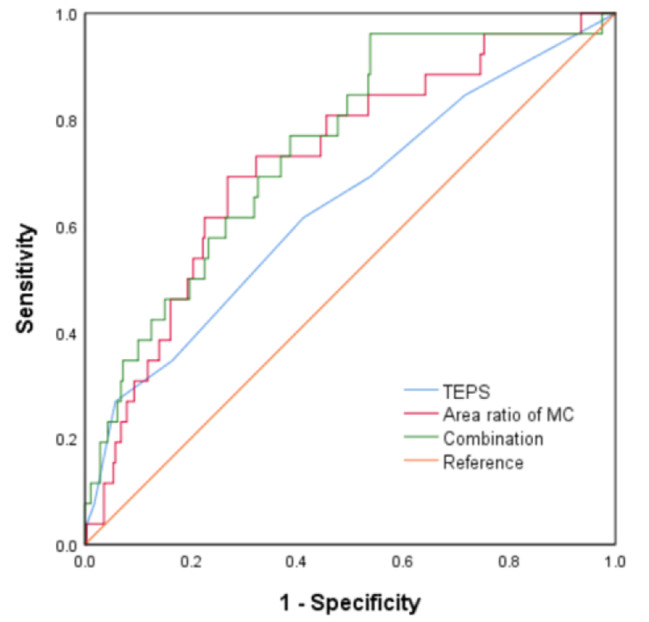
ROC curve analysis TEPS and area ratio of MC

ROC curve analysis was performed for the TEPS and the area ratio of MC. The results showed that the area ratio of MC had moderate diagnostic value for SSI (AUC: 0.722; CI: 0.621–0.824). By using the Youden index, the cutoff value was determined to be 24.62% (sensitivity: 69.2%; specificity: 73.1%). However, the TEPS had poor diagnostic value for SSI (AUC: 0.641; CI: 0.522–0.759), and the combined diagnosis of the two did not significantly improve the diagnostic value (AUC: 0.747; CI: 0.653–0.842) (Fig. 3) (Table 4).

**Table 4:** Diagnostic performance of the variables

Variables	AUC	Youden	Cutoff	Sensitivity (%)	Specificity (%)
Ratio of MC	0.722 (0.621–0.824)	0.423	24.62	69.2	73.1
TEPS	0.641 (0.522–0.759)	0.245	5.5	62.5	62.0
Combination	0.747 (0.653–0.842)	0.424	-	96.2	46.2

## Discussion

The intervertebral disc is in a relatively vacuum state and relies on the permeability of the endplate to provide nutrition. Studies have shown that the infection pathway begins with intervertebral disc protrusion and annulus fibrosus injury, and the repair process involves neovascularization, allowing bacteria to enter the intervertebral disc through fissures, leading to slow-developing infection of the intervertebral disc and vertebral endplate. With the release of inflammatory mediators, the infection can spread through the vertebral endplate to the adjacent bone marrow, causing changes in MRI signals. This subclinical infection may result in systemic inflammatory response and postoperative complications [23–25]. To validate this finding, we linked MC to SSI and retrospectively analyzed 646 patients who underwent single-level lumbar spine surgery to explore in detail the correlation between MC (including presence, type, location, area, TEPS) and SSI. The severity of MC was quantified with MRI, defined as the area ratio of MC. The predictive value of area ratio of MC and TEPS for SSI was compared.

### Modic changes and SSI

Currently, research on the potential correlation between MC and SSI is limited and controversial. To verify the relationship between MC and SSI, Ken Ninomiya et al. examined data from 2721 patients undergoing lumbar laminectomy without discectomy in five hospitals, Patients who developed postoperative discitis following laminectomy (Group D) and a 4:1 matched cohort (Group C) were included. The results exhibited that type 1 MC was an independent risk factor for postoperative purulent intervertebral disc inflammation, and there was no statistically significant difference in disc generation grading and endplate loss between the two groups. However, Pradip et al. conducted a retrospective analysis of 1124 patients undergoing spinal surgery, comprehensively investigating a probable association between preoperative MC, TEPS, and SSI. The results showed that the presence of preoperative MC and TEPS > 6 were independent risk factors for surgical site infection, independent of the type and location of MC. The novelty of our study lies in quantifying the severity of MC and predicting SSI through the area ratio of MC measured by MRI. The results indicate that the area ratio of MC is an independent risk factor for SSI. The area ratio of MC is a moderate predictor of SSI (AUC = 0.722). For every 1% increase, the incidence of postoperative SSI increases by 10.3% (OR = 1.103, *P* = 0.001). When the incidence rate exceeds 24.62%, attention should be paid to the occurrence of SSI. Furthermore, consistent with the findings of Pradip et al., this study found that TEPS is also an independent risk factor for SSI (OR = 1.382, *P* = 0.009). However, the diagnostic efficacy of TEPS for SSI (AUC = 0.641) is lower than that of MC area ratio. This indicate that the area ratio of MC can more directly evaluate the relationship between MC and SSI.

### Types and locations of Modic changes

Previous studies have indicated that Type 1 MC is associated with inflammatory reactions, Type 2 MC represents fatty marrow conversion, and Type 3 MC involves fatty marrow deposition replaced by sclerotic bone. Therefore, many studies have excluded Type 2 and 3 and focused only on Type 1 [17, 26]. However, to provide a more comprehensive understanding, this study included all three types to assess their correlation with SSI. The results showed no significant difference in the distribution of SSI among the three types. MC is a continuous process, where various types can transform from each other or even disappear [21]. This suggests that the different types of MC may be interrelated and collectively influence the occurrence of SSI. Additionally, since the location of MC within the endplate varies and the exposure opportunities differ in lumbar spine surgery, we also explored the correlation between the location of MC (including anterior, central, posterior, or involving the entire endplate) and SSI. The results showed no significant correlation between the location of MC within the endplate and SSI, which is consistent with the findings of Pradip et al. [18]. This result may suggest that the cause of SSI in MC patients may be related to systemic inflammatory response rather than direct bacterial colonization. This provides valuable insights for further investigation into the differences in inflammatory markers and pro-inflammatory factors between patients with and without MC.

### The area ratio of Modic changes and SSI

Area ratio is often used as an indicator to evaluate the severity of lesions. Therefore, in this study, we attempted to use the area ratio of MC to assess its correlation with SSI. Previous studies have shown a positive correlation between endplate area and sagittal area in MC, indicating that the spread of MC within the endplate and vertebral body is synchronous [27]. Therefore, this study only included the sagittal plane MC area, which is more convenient and efficient. By using the ratio of the sagittal plane MC area to the corresponding vertebral body area as a reference index, individual differences were eliminated, making the conclusions more reliable. Our results indicate that when the area ratio of MC exceeds 24.62%, there is a risk of postoperative SSI in patients. Perioperative use of antibiotics can be considered to reduce the incidence of postoperative SSI. Interestingly, previous studies have explored the correlation between MC area and lower back pain, and the results showed a positive correlation between the area of Type 1 MC and changes in lower back pain symptoms [28]. After antibiotic treatment, lower back pain symptoms in patients with MC were relieved, and the MC area significantly decreased [29], Conventional treatment methods are often ineffective [30]. Therefore, preoperative antibiotic use can not only prevent postoperative SSI in patients with MC but also potentially improve surgical outcomes. According to previous research findings, antibiotic treatment for MC patients requires a long-term process to alleviate lower back pain symptoms and even reduce MC area, but the treatment duration ranges from 90 to 100 days [29, 31]. Due to the controversy over the duration of antibiotic use, combined with the results of this study, it is recommended that to use antibiotics to reduce the area ratio below the cut-off value (24.62%) before surgery. At the same time, it is necessary to consider the financial burden of the patient and the side effects of the drug [32].

### The total endplate score and SSI

TEPS was first proposed by Rajasekaran et al. To study disc spread patterns by injecting Gadodiamide and obtaining serial postcontrast MRI images. The upper and lower endplates of each lumbar disc in 73 individuals were analyzed, and endplate injuries were graded. Quantification of endplate injury using TEPS and its correlation with the severity of degenerative intervertebral disc disease. According to the TEPS criteria, the presence of MC in a single endplate is scored greater than 4 [33]. Therefore, TEPS was also included in this study to explore its correlation with SSI. The results suggest that TEPS is an independent risk factor for SSI, which further confirms the correlation between MC and SSI. However, ROC curve analysis showed that its diagnostic value was lower than that of area ratio, and the combined diagnosis of the two did not significantly improve the diagnostic value.

### Comorbidities and SSI

This study also explored the correlation between several common comorbidities and SSI. In the univariate analysis, there was a significant difference in the prevalence of diabetes between the two groups, but after conducting a multivariate regression analysis and excluding various confounding factors, it was suggested that diabetes was not significantly correlated with SSI. This may be because during the treatment process, we controlled the blood sugar and other comorbidities of perioperative patients within a relatively stable range, thereby reducing the risk of postoperative SSI.

### Limitations

There are some limitations to this study. Firstly, the sample size for SSI was small due to its low incidence rate. Secondly, this study was single-center, and future research could consider multi-center studies to further confirm the conclusions. Thirdly, this study did not compare the differences in inflammatory markers and cytokines between MC and non-MC, in order to further confirm the systemic inflammatory response of patients with MC. Fourthly, due to being a retrospective study, tissue culture was not performed to isolate P. acnes to further confirm its correlation with MC. In the future, this experiment can be attempted and targeted antibiotics can be used to prevent SSI and lower back pain based on antimicrobial susceptibility test.

## Conclusion

The area ratio of Modic changes has predictive value for postoperative surgical site infection in lumbar spine surgery. When the rate of MC is greater than 24.62%, we should be alert to the occurrence of SSI.

## Data Availability

No datasets were generated or analysed during the current study.

## Abbreviations

MC: Modic changes
SSI: Surgical site infection
TEPS: Total endplate score
CDC: Centers for Disease Control and Prevention
ROC: Receiver operating characteristic
ASA: American Society of Anesthesiologists
CHD: Coronary heart disease
COPD: Chronic obstructive pulmonary disease
AUC: Area under the curve
MRI: Magnetic resonance imaging
T1WI: T1-weighted images
T2WI: T2-weighted images
P. acnes: Propionibacterium acnes
